# The roles of serum Th1, Th2, and Th17 cytokines in patients with chronic urticaria: a systematic review and meta-analysis

**DOI:** 10.3389/falgy.2025.1673041

**Published:** 2025-10-03

**Authors:** Jingwen Xue, Chinghsuan Sun, Mai Shi, Bingyu Li, Yi Zhao

**Affiliations:** 1 Department of Dermatology, Beijing Tsinghua Changgung Hospital, School of Clinical Medicine, Tsinghua Medicine, Tsinghua University, Beijing, China; 2 Photomedicine Laboratory, Institute of Precision Medicine, Tsinghua University, Beijing, China

**Keywords:** chronic urticaria, serum biomarkers, TNF-α, IL-17, T lymphocytes

## Abstract

**Objectives:**

To conduct a systematic review and meta-analysis to identify Th1-, Th2, and Th17 related serum biomarkers that reflect disease activity in chronic urticaria (CU), thereby enhancing the assessment of disease activity in both trials and clinical practice.

**Methods:**

Systematic searches of PubMed, EMBASE, and Web of Science were conducted through November 2024 to identify articles reporting the associations between CU and serum biomarkers. Serum Th1, Th2, and Th17 related biomarkers were identified in CU patients and correlated with disease severity and patient characteristics (ex. Age, sex, and comorbidities). The study quality was assessed using the National Heart, Lung, and Blood Institute Quality Assessment Tool for case-control studies. Meta-analysis was performed using the random-effects model with Hedges' g to pool standardized mean differences (SMDs). For meta-analysis, data were included for biomarkers reported in at least four studies with available means and standard deviations (SDs). Data reported as medians with ranges or interquartile ranges (IQRs) were evaluated for skewness. If the data were found to be significantly skewed, the means and SDs were not calculated. Conversely, if the data were not skewed, the means and SDs were estimated using validated methods.

**Results:**

A total of 6,013 studies were screened, of which 50 were included, reporting 22 serum Th1, Th2, and Th17 related cytokines. Meta-analyses revealed significant pooled standardized mean differences (SMDs) for serum TNF-α and IL-17.

**Conclusions:**

Serum TNF-α and IL-17 levels are significantly increased in patients with CU compared to healthy age- and sex-matched controls. These findings have the potential to influence clinical guidelines for the diagnostic workup of CU to include testing the serum levels of TNF-α and IL-17.

## 1 Introduction

Chronic urticaria (CU) is a common and debilitating mast cell-driven skin disease that persists for over 6 weeks, and is characterized by wheals, angioedema, or both (1). The underlying pathophysiology of CU remains poorly understood. While a central role for mast cells has traditionally been proposed, growing evidence suggests that immune dysregulation mediated by T lymphocytes also plays a significant role (2).

The activity of T lymphocytes largely depends on the differentiation of CD4^+^ T cells into distinct functional subsets, such as Th1, Th2, Th17, and T follicular helper (Tfh) cells, mediated by specific cytokine production (3). A complex network of cytokines released by Th cells into the peripheral circulatory microenvironment are thought to influence the immune response in CU (4–7).

Th1 cells produce cytokines such as IL-1β, IL-2, IL-12, IL-18, IFN-γ, and tumor necrosis factor (TNF), and are involved in cell-mediated pro-inflammatory responses (8). Th2 cells secrete cytokines like IL-4, IL-5, IL-6, IL-9, IL-10, IL-13, IL-17E, IL-31, and IL-33, which can inhibit Th1 cytokine production (9). Th2 cytokines are involved in antibody responses, particularly IgE production, and activate eosinophils and mast cells (10, 11). The imbalance between Th1 and Th2 has long been considered a potential mechanism in urticaria (12, 13). Th17 cells, which produce IL-17, IL-17A, IL-17F, IL-21, IL-22, IL-23, IL-25, and transforming growth factor (TGF)-β, are implicated in the pathogenesis of autoimmune and allergic diseases (14, 15).

In this study, we aimed to better characterize the helper T-cell immune phenotype in CU patients. The clinical roles of serum Th1, Th2, and Th17 cytokines in CU patients are discussed in this systematic review.

## 2 Methods

### 2.1 Literature search strategy

We systematically searched PubMed, EMBASE, and Web of Science from inception to November 2024. The protocol was registered in PROSPERO (CRD42024608505). Eligible studies quantified serum cytokines in chronic urticaria (CU). The search strategy combined Medical Subject Headings and free-text terms for CU (“Chronic Urticaria”, “Chronic Spontaneous Urticaria”, “Idiopathic Chronic Urticaria”, “Autoimmune Urticaria”, and “Chronic Autoimmune Urticaria”) with terms for biomarkers (“Biomarkers”, “Biologic Markers”, “Clinical Marker”, “Serum Markers”, “Cytokines”, “Chemokines”, and “Inflammation Mediators”).

### 2.2 Eligibility criteria

We included original research articles that compared serum levels of Th1-, Th2, and Th17 related cytokines between CU patients and healthy controls and examined associations with disease presence, activity, or severity. We excluded case reports, animal studies, editorials, letters, and studies that exclusively measured biomarkers in tissue biopsies, lymphocyte subsets, or genetic polymorphisms.

### 2.3 Study selection and data extraction

The outcome was the identification and correlation of cytokine levels with CU. Two reviewers (JWX and CHS) independently screened titles, abstracts, and full texts using EndNote. Discrepancies were resolved by a third reviewer (YZ). Data were extracted and verified by JWX and CHS. The author lists and publication years were screened to exclude possible duplicate or overlapping studies. The extracted variables included study characteristics, patient demographics (age and sex), biomarker levels, and reported associations with disease activity or severity, along with *p*-values. If any data were incomplete or unclear, the corresponding authors of the selected studies were contacted for further detail.

### 2.4 Quality evaluation

Study quality was assessed using the National Heart, Lung, and Blood Institute (NHLBI) Quality Assessment Tools, which are applicable to cohort, cross-sectional, case-control, and case series studies. This systematic review was conducted in accordance with the Preferred Reporting Items for Systematic Reviews and Meta-Analyses (PRISMA) guidelines. JWX and CHS drafted the manuscript. All authors contributed to the development of the selection criteria and the data extraction protocols. All the authors have read and approved the final manuscript.

### 2.5 Statistical analysis

Meta-analyses were performed using the random-effects model with Hedges' g to account for small-sample bias and between-study heterogeneity. Pooled standardized mean differences (SMDs) were calculated for biomarkers reported in at least four studies with available means and standard deviations (SDs). Studies were excluded from the meta-analysis if: (1) biomarker levels were reported as undetectable; (2) SDs were not reported or calculable; (3) data were presented only as medians with ranges or interquartile ranges (IQRs) and were determined to be skewed upon inspection. Data skewness was evaluated for studies that reported medians with IQRs or ranges. If not significantly skewed, means and SDs were estimated using validated methods (16). When applicable, 95% confidence intervals or ranges were used to back-calculate the SDs.

## 3 Results

**Figure 1:**
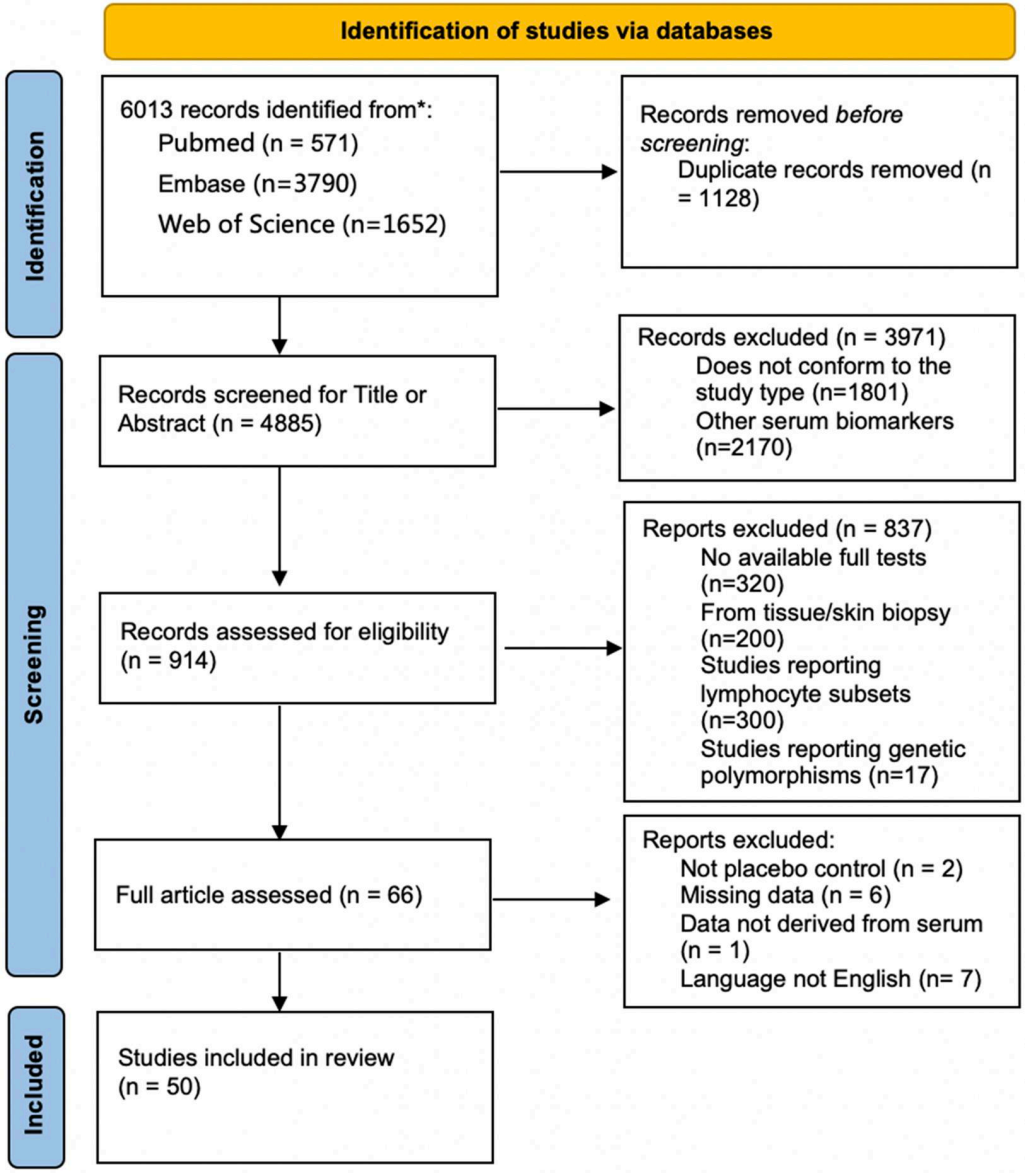
PRISMA (Preferred Reporting of Items in Systematic Reviews and Meta-Analysis) flowchart depicting the identification, screening, and inclusion of the studies included.

A total of 6,013 studies were screened, of which 50 were included, reporting 22 serum Th1-, Th2, and Th17 related cytokines (Figure 1: PRISMA flow diagram). Meta-analyses revealed significant pooled SMDs for serum TNF-α and IL-17.

### 3.1 Th1 cytokines

**Figure 2:**
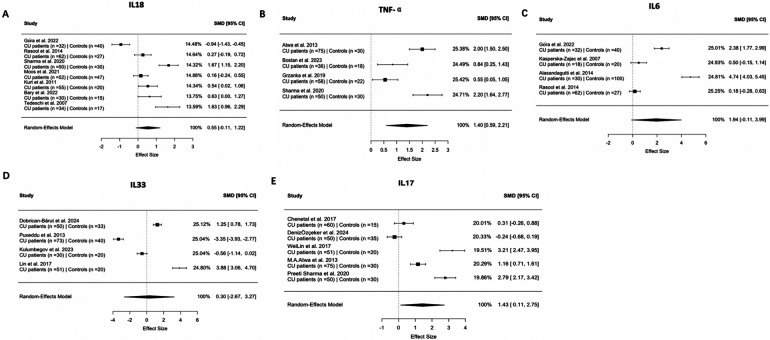
Forest plot for the comparison of **(A)** serum IL-18, **(B)** TNF-α, **(C)** IL-6, **(D)** IL-33, and **(E)** IL-17 cytokines in chronic urticaria patients vs. healthy control. The forest plot was generated using the random-effects model with Hedges' g to account for small-sample bias and between-study heterogeneity. SMD = standardized mean difference in the concentrations of serum biomarkers between groups.

IL-18 was the most frequently assessed Th1-related cytokine in nine studies. Of these, five studies reported elevated IL-18 levels in CU patients compared to controls, one study described a reduction, and three studies found no significant difference. A pooled analysis of seven studies involving 511 participants yielded a standardized mean difference (SMD) of 0.55 (95% CI, −0.11–1.22), indicating considerable variation among study results (Figure 2A). TNF-α levels were examined in 11 studies. Eight studies identified increased serum levels in CU patients, while the remaining three reported no association. Meta-analysis of four studies with 319 participants showed a significant difference, with a pooled SMD of 1.40 (95% CI, 0.59–2.21) (Figure 2B). IFN-γ levels were evaluated in five studies. Three of these studies observed higher levels in CU patients than in healthy controls, and two did not find a statistically significant difference. IL-12 was reported in three studies, all of which documented elevated levels in CU patients relative to the control groups. IL-2 levels were measured in three studies. One study identified increased IL-2 concentrations in CU patients, while two reported no such association (Table 1). IL-1β was included in one study, which observed higher serum levels in the CU group than in the controls (Table 1).

**Table 1:** Th1 cytokines results.

Th1 Cytokines	Study (author-yr)	Overall association	Sample size of CU patients	Sample size of controls	Value in CU patients (mean ± SD) pg/ml/*P* value	Value in controls (mean ± SD) pg/ml	Age of CU patients (mean ± SD, yrs)	Age of controls (mean ± SD, yrs)	Country
IL-1β	Santos et al. (17)	Increased	29	33	NA(*P* < 0.001)	NA	42.5 (20–66)	35.5 (23–57)	Brazil
IL-2	Piconi et al. (18)	No association	19	15	NA	NA	39.15 (23–61)	38.7 (23–54)	Italy
IL-2	Chen et al. (6)	Increased		15	747.4 (414.2–1,205.7)/0.001	424.7 (218.3–545.2)	NA	24.6 (22–29)	China
IL-2	Grieco et al. (19)	No association	8	4	4.8/NA	NA	57 ± 4	40 ± 8	Italy
IL-12	Petrola et al. (20)	Increased	20	30	17.7 pgr/ml, *P* < 0.05	0 pgr/ml	33.0 (6 14.8)	NA	Venezuela
IL-12	Ene et al. (21)	Increased	42	40	38.0 ± 14.1(*p* < 0.001)	17.9 ± 4.7	31.3 *±* 10.8	29.8 *±* 10.2	Romania
IL-12	Santos et al. (17)	Increased	29	33	NA(*P* < 0.01)	NA	42.5 (20–66)	35.5 (23–57)	Brazil
IL-18	Gora et al. (22)	Decreased	32	40	115.8 (102.5–129.5)/<0.001 (115.9424 ± 20.9529)	133.25 (122.95–142.15) (132.7532 ± 14.7635)	11.21 (8–14.42)	11 (7–14)	Poland
IL-18	Rasool et al. (23)	No association	62	27	62.95 ± 36.09/0.24	54.35 ± 18.45	28 (7–65)	NA	India
IL-18	Sharma et al. (24)	Increased	50	30	501.41 ± 208.98, *P* < 0.05	218.39 ± 39.83	33.84 ± 6.816	33.20 ± 7.014	India
IL-18	Moos et al. (25)	No association	52	47	33.68 (30.92–38.47), *P* = 0.8, (34.3981 ± 5.7564)	32.72 (31.13–36.76) (33.5877 ± 4.3055)	40.6 ± 12.4 (19–68)	41.6 ± 12.1 (19–64)	Poland
IL-18	Varghese et al. (26)	Increased	45	45	144.3 (118.4–197.3), *P* < .001	104.3 (89.0–115.8)	35.91 (9.30), *P* = 0.66	35.04 (9.20)	India
IL-18	Kurt et al. (27)	Increased	55	20	290.0 ± 178.2, *P* = 0.031	202.2 ± 96.8	40.3 ± 12.3	37.7 ± 9.8	Turkey
IL-18	Bary et al. (28)	Increased	30	15	214.9 ± 167.3 pg/ml, *P* = 0.035	115.6 ± 121.1 pg/ml	35.70 ± 13.87 (16–81)	NA	Egypt
IL-18	Tedeschi et al. (29)	No association	34	17	246.47 ± 18.40 pg⁄ml, NS	213.88 ± 22.24 pg⁄ml	45.9 ± 2.6 years	43.9 ± 3.3	Italy
IL-18	Puxeddu et al. (30)	Increased	73	40	NA (*P* = 0.0003)	NA	41 ± 16, (14–89)	50 ± 5, (25–89)	Italy
TNF-α	Chen et al. (6)	Increased	60	15	1.6 (0.8–99.1)/0.004	0.0 (0.0–0.0)	NA	24.6 (22–29)	China
TNF-α	Tekin et al. (31)	No association	31	56	59.57 (35.51–500.46)/NA	64.83 (32.84–613.41)	NA	NA	Turkey
TNF-α	Trinh et al. (32)	Increased	191	89	NA	NA	40 (19–60)	38 (26–54)	Korea
TNF-α	Grieco et al. (19)	No association	8	4	9.11/NA	NA	57 ± 4	40 ± 8	Italy
TNF-α	Atwa et al. (33)	Increased	75	30	17.93 ± 6.05, *P* = 0.004	6.87 ± 3.73	31.8 ± 10.3	29.7 ± 9.1	Egypt
TNF-α	Bostan et al. (34)	Increased	36	18	156.7 (33.08–392), *P* < 0.01, (196.3929 ± 277.114)	1.8 (1.5–2.91) (2.0923 ± 1.1343)	38.5 ± 11.9	38 (30–47)	Turkey
TNF-α	Tedeschi et al. (35)	No association	40	12	NA	NA	NA	NA	Italy
TNF-α	Grzanka et al. (36)	Increased	58	22	18.25 (17.04–19.62), *P* < 0.05, (18.3065 ± 1.9614)	16.89 (16.45–18.40) (17.274 ± 1.5454)	39 (21–45)	NA	Poland
TNF-α	Sharma et al. (24)	Increased	50	30	455.54 ± 253.54, *P* < 0.05	8.498 ± 3.644	33.84 ± 6.816	33.20 ± 7.014	India
TNF-α	Santos et al. (17)	Increased	29	33	NA(*P* < 0.01)	NA	42.5 (20–66)	35.5 (23–57)	Brazil
TNF-α	Piconi et al. (18)	Increased	19	15	NA(CIU vs. HCs, *p*< 0.0001)	NA	39.15 (23–61)	38.7 (23–54)	Italy
IFN-γ	Chen et al. (6)	Increased	60	15	368.9 (223.5–433.5)/0.004	250.8 (19.6–404.8)	NA	24.6 (22–29)	China
IFN-γ	Caproni et al. (37)	No association	68	20	NA	2.4 ± 2.2	43 (19–81)	39 (21–74)	Italy
IFN-γ	Grieco et al. (19)	Increased	8	4	8.37/<0.05	NA	57 ± 4	40 ± 8	Italy
IFN-γ	Piconi et al. (18)	No association	19	15	NA	NA	39.15 (23–61)	38.7 (23–54)	Italy
IFN-γ	Alasandagutti et al. (38)	Increased	30	100	80.762 ± 62.056/<0.0001	24.79 ± 21.84	34.1	NA	India

Yr, year; Yrs, years; SD, standard deviation; CU, chronic urticaria.

### 3.2 Th2 cytokines

Among the Th2 cytokines, IL-6 was the most frequently studied, as reported in 18 articles. Fifteen studies observed elevated IL-6 levels in CU patients compared to healthy controls, while three studies reported no association. A meta-analysis based on four studies involving 329 participants indicated a pooled SMD of 1.94 (95% CI, −0.11–3.99), suggesting a trend toward elevation without reaching statistical significance (Figure 2C). IL-4 levels were investigated in eight studies. Two studies documented increased IL-4 levels in CU patients, two reported reduced levels, and four found no significant association (Table 2). IL-5 expression was examined in two studies. One study recorded higher levels in CU patients than in controls, and another found no difference (Table 2). IL-10 was reported in nine studies. Among these, five studies observed increased levels in CU patients, one study noted a decrease, and three reported no association (Table 2). IL-13 levels were evaluated in four studies. Three studies reported elevated IL-13 levels in CU patients, and one study showed no statistically significant difference (Table 2). IL-31 was included in seven studies. Four studies identified higher IL-31 levels in CU cases than in controls, one study reported reduced levels, and two studies found no association (Table 2). IL-33 has also been reported in seven studies. Two studies observed increased IL-33 levels in CU patients, two reported lower levels, and three studies did not identify a significant difference (Table 2). A meta-analysis of four studies with 317 participants revealed a pooled SMD of 0.30 (95% CI, −2.67–3.27) (Figure 2D). IL-9 levels were measured in two studies, both of which found no significant differences between CU patients and controls (Table 2). IL-24 was investigated in one study that reported higher levels in the CU group than in the control group (Table 2).

**Table 2:** Th2 cytokines.

Th2 Cytokines	Study (author-yr)	Overall association	Sample size of CU patients	Sample size of controls	Value in CU patients(mean ± SD) pg/ml/*P* value	Value in controls(mean ± SD) pg/ml	Age of CU patients(mean ± SD, yrs)	Age of controls(mean ± SD, yrs)	Country
IL-4	Degirmenci et al. (39)	Decreased	40	20	NA/0.04	NA	38.2 ± 10.4	36.5 ± 5.45	Turkey
IL-4	Chen et al. (6)	No association	60	15	3.1 (1.5–7.6)/0.021	2.2 (0.5–3.9)	NA	24.6 (22–29)	China
IL-4	Hoşgören-Tekin et al. (31)	Decreased	31	56	101.1 (57.8–418.6)/0.001	138.85 (70.5–508.4)	NA	NA	Turkey
IL-4	Ferrer et al. (40)	Increased	60	25	1.03/0.028	0.2	44.39 ± 2.91	40 ± 6.49	USA
IL-4	Caproni et al. (37)	No association	68	20	NA	0.01 ± 0.06	43 (19–81)	39 (21–74)	Italy
IL-4	Grieco et al. (19)	No association	8	4	0.04/NA	NA	57 ± 4	40 ± 8	Italy
IL-4	Mohamed et al. (41)	Increased	25	10	18,300 ± 14,700/0.008	5,100 ± 4,100	NA	NA	Egypt
IL-4	Zheng et al. (42)	No association	28	28	NA/>0.05	NA	35.6 ± 6.1	35.8 ± 7.9	China
IL-5	Chen et al. (6)	Increased	60	15	8.5 (5.6–18.6)/0.018	5.6 (2.6–9.5)	NA	24.6 (22–29)	China
IL-5	Hoşgören-Tekin et al. (31)	No association	31	56	61.65 (34.1–471.4)/NA	73.1 (30.9–660.3)	NA	NA	Turkey
IL-6	Chen et al. (6)	Increased	60	15	167.0 (126.6–261.2)/0.004	107.8 (29.4–171.6)	NA	24.6 (22–29)	China
IL-6	Trinh et al. (32)	Increased	191	89	NA	NA	40 (19–60)	38 (26–54)	Korea
IL-6	´Gora et al. (22)	Increased	32	40	13.91 (11.32–15.71)/<0.001(13.6,287 ± 3.4068)	7.42 (6.21–8.45)(7.3561 ± 1.7224)	11.21 (8–14.42)	11 (7–14)	Poland
IL-6	Ucmak et al. (43)	Increased	50	33	46.57/<0.001	20.34	35 (18–55)	36 (20–60)	Turkey
IL-6	Amin et al. (44)	Increased	40	40	NA/<0.001	NA	NA	NA	Egypt
IL-6	Grieco et al. (19)	Increased	8	4	6.2/<0.05	NA	57 ± 4	40 ± 8	Italy
IL-6	Kasperska-Zajac et al. (45)	Increased	58	22	3.32/<0.0001	0.69	38 (24–52)	NA	Poland
IL-6	Grzanka et al. (46)	Increased	58	22	3.95 (1.98–9.2)/<0.0001	1.0 (0.43–1.58)	39 (21–45)	NA	Poland
IL-6	Valerieva et al. (47)	Increased	45	NA	1.91 ± 0.50/<0.001	0.03 ± 0.02	NA	NA	Bulgaria
IL-6	Kasperska-Zajac et al. (48)	Increased	8	20	NA/0.0026	NA	37 (28–43)	NA	Poland
IL-6	Kasperska-Zajac et al. (49)	Increased	18	20	1 (0.12–2.22)/0.033(1.1227 ± 1.6894)	0.39 (0.05–0.97)(0.4763 ± 0.734)	30.5 (19–37)	NA	Poland
IL-6	Kasperska-Zajac et al. (50)	Increased	58	30	1.85/<0.001	1.1	40	NA	Poland
IL-6	Rajappa et al. (51)	Increased	45	45	NA/<0.0001	NA	NA	NA	India
IL-6	Alasandagutti et al. (38)	Increased	30	100	39.37 ± 11.06/<0.0001	7.175 ± 4.81	34.1	NA	India
IL-6	Rasool et al. (23)	No association	62	27	0.82 ± 4.6/0.44	0.12 ± 1.7	28 (7–65)	NA	India
IL-6	Grzanka et al. (52)	No association	17	16	11.64 (10.34–11.93)/<0.0001	4.95 (4.05–5.7)	42 (29–45)	NA	Poland
IL-6	Bostan et al. (34)	No association	36	18	1.37 (0.90–3.93)	1.0 (0.7–1.6)	38.5 ± 11.9	38 (30–47)	Turkey
IL-6	Santos et al. (17)	Increased	29	33	NA(*P* *<* 0.001)	NA	42.5 (20–66)	35.5 (23–57)	Brazil
IL-9	Bhatia et al. (53)	No association	95	42	1,607 ± 1,182.5/0.082	1,838.70 ± 929.89	33.77 ± 8.07	NA	India
IL-9	Zheng et al. (42)	No association	28	28	NA/>0.05	NA	35.6 ± 6.1	35.8 ± 7.9	China
IL-10	Degirmenci et al. (39)	Decreased	40	20	NA/0.04	NA	38.2 ± 10.4	36.5 ± 5.45	Turkey
IL-10	Chen et al. (6)	No association	60	15	3.1 (2.4–5.8)/NA	3.0 (1.2–5.2)	NA	24.6 (22–29)	China
IL-10	Hoşgören-Tekin et al. (31)	No association	31	56	113.57 (73.24–738.31)/NA	119.52 (77.82–782.9)	NA	NA	Turkey
IL-10	Trinh et al. (32)	Increased	191	89	NA	NA	40 (19–60)	38 (26–54)	Korea
IL-10	Grieco et al. (19)	No association	8	4	11.01/NA	NA	57 ± 4	40 ± 8	Italy
IL-10	Valerieva et al. (47)	Increased	45	NA	5.91 ± 0.48/<0.001	0.86 ± 0.51	NA	NA	Bulgaria
IL-10	Moos et al. (25)	Increased	52	47	4.25 (2.57–5.49),*P* = 0.04	3.36 (1.59–4.25)	40.6 ± 12.4 (19- 68)	41.6 ± 12.1 (19- 64)	Poland
IL-10	Santos et al. (17)	Increased	29	33	NA(*P* < 0.001)	NA	42.5 (20–66)	35.5 (23–57)	Brazil
IL-10	Piconi et al. (18)	Increased	19	15	NA(CIU vs.HCs,*p* = 0.002)	NA	39.15 (23–61)	38.7 (23–54)	Italy
IL-13	Chen et al. (6)	Increased	60	15	19.6 (13.6–31.2)/0.008	15.0 (8.1–19.7)	NA	24.6 (22–29)	China
IL-13	Hoşgören-Tekin et al. (31)	No association	31	56	9.3 (5.80–58.47)/NA	9.61 (5.22–55.82)	NA	NA	Turkey
IL-13	Bae et al. (54)	Increased	84	43	508.5 ± 51.2/0.001	200.7 ± 13.3	38.2 ± 12.7	30.4 ± 9.3	Korea
IL-13	Caproni et al. (37)	Increased	68	20	NA	2.4 ± 2.2	43 (19–81)	39 (21–74)	Italy
IL-24	Laurence de Montjoye et al. (55)	Increased	69	23	NA, *P* < 0.05	NA	NA	NA	Belgium
IL-31	Lin et al. (56)	Increased	51	20	27.79 ± 3.02 ng/L, *P* < 0.001	18.78 ± 1.71 ng/L	28 ± 13, *p* = 0.285	32 ± 14	China
IL-31	Bostan et al. (34)	No association	36	18	743.2 (121.3–3,447)	213.1 (9–633.6)	38.5 ± 11.9	38 (30–47)	Turkey
IL-31	Boyvadoglu et al. (57)	Decreased	30	20	65.30 (46.39–89.14),*p* = 0.001(67.0575 ± 33.2775)	169.57 (115.8–237.27)(174.5813 ± 96.9144)	39.83 ± 11.67, *P* = 0.008	31.85 ± 6.77	Turkey
IL-31	Băruta et al. (58)	Increased	50	38	NA, *p* < 0.0001	NA	50.14 ± 16.10	44.32 ± 9.23	Romania
IL-31	Raap et al. (59)	Increased	46	26	NA, *P* < 0.001	NA	NA	43.8 ± 16.5	Germany
IL-31	Chaowattanapanit et al. (60)	Increased	65	31	(252.4 ± 115.5, *P* < 0.001	36.3 ± 10.7 pg/ml	43 ± 15	44 ± 18	Thailand
IL-31	Hoşgören-Tekin et al. (31)	No association	31	56	43.69 (18.23–298.67)/NA	47.33 (24.99–436.21)	NA	NA	Turkey
IL-33	Băruta et al. (61)	Increased	50	33	220.67 ± 201.17, *P* < 0.0001	21.70 ± 22.68	50.14 ± 16.10	44.32 ± 9.23	Romania
IL-33	Puxeddu et al. (30)	No association	73	40	575.3 ± 105.6, NS	1,189 ± 271.5	41 ± 16, (14–89)	50 ± 5,(25–89)	Italy
IL-33	Kulumbegov et al. (62)	No association	30	20	29.74 ± 5.02, *P* = 0.093	33.2 ± 7.43	37.6 ± 17.57	44.45 ± 14.83	Israel
IL-33	Lin et al. (56)	Increased	51	20	45.53 ± 4.32 ng/L, *P* < 0.001	30.09 ± 2.69 ng/L	28 ± 13, *p* = 0.285	32 ± 14	China
IL-33	Hoşgören-Tekin et al. (31)	Decreased	31	56	154.89 (85.82–1,142.36)/0.038	200.53 (108.78–1,381.42)	NA	NA	Turkey
IL-33	Valerieva et al. (47)	Decreased	45	NA	0.89 ± 0.41/0.005	5.04 ± 1.02	NA	NA	Bulgaria
IL-33	Zheng et al. (42)	No association	28	28	NA/>0.05	NA	35.6 ± 6.1	35.8 ± 7.9	China

Yr, year; Yrs, years; SD, standard deviation; CU, chronic urticaria.

### 3.3 Th17 cytokines

A meta-analysis of five studies involving 416 participants demonstrated a significant elevation in serum IL-17 levels among patients with chronic urticaria, with a pooled SMD of 1.43 (95% CI, 0.11–2.75) (Figure 2E). IL-17 was evaluated across nine studies; five of them identified increased levels in CU cases compared to controls, while the remaining four studies reported no statistically significant association (Table 3). Two studies investigated IL-17A concentration. Among them, one study documented elevated serum IL-17A levels in CU patients relative to controls, and another study found no significant difference between groups (Table 3). IL-17F was measured in one study, which indicated no notable difference in serum levels between CU and control cohorts (Table 3). IL-21 was reported in one study that observed higher levels in CU patients than in healthy controls (Table 3). IL-22 was included in one publication, with results showing no measurable differences between groups (Table 3). Five studies assessed the IL-23 levels. Of these, three studies observed elevated IL-23 concentrations in CU patients and two studies did not detect a meaningful difference when compared with controls (Table 3). IL-25 was included in one study that reported increased serum levels in the CU group compared to the control group (Table 3).

**Table 3:** Th 17 cytokines.

Th17 Cytokines	Study (author-yr)	Overall association	Sample size of CU patients	Sample size of controls	Value in CU patients(mean ± SD) pg/ml/*P* value	Value in controls(mean ± SD) pg/ml	Age of CU patients(mean ± SD, yrs)	Age of controls(mean ± SD, yrs)	Country
IL-17	Chen et al. (6)	No association	60	15	1.3 (0.4–2.7)/NA(1.4766 ± 1.747)	0.6 (0.0–2.1)(0.9267 ± 1.7176)	NA	24.6 (22–29)	China
IL-17	Özçeker et al. (63)	No association	50	35	3.98 ± 3.88/3.1 (2.6–4.6), *p* = 0.063	4.85 ± 2.96/3.9 (3.0–6.3)	NA	NA	Turkey
IL-17	Moghadam K et al. (64)	Increased	60	30	NA	209.47(sd:106.55)	NA	NA	Iran
IL-17	Bostan et al. (34)	No association	36	18	47.7 (8.2–268.8)	100 (15.8–639)	38.5 ± 11.9	38 (30–47)	Turkey
IL-17	Lin et al. (56)	Increased	51	20	256.71 ± 25.07 ng/L, *P* < 0.001	181.79 ± 16.62	28 ± 13, *p* = 0.285	32 ± 14	China
IL-17	Atwa et al. (33)	Increased	75	30	35.51 ± 31.14 pg/ml, *P* < 0.001	4.60 ± 1.38	31.8 ± 10.3	29.7 ± 9.1	Egypt
IL-17	Grzanka et al. (65)	Increased	52	21	21.97 (20.92–24.98/18.85–62.73) pg/ml, *p* < 0.001	19.88 (18.85–20.92/17.82–59.16) pg/ml	38 (24–50)	NA	Poland
IL-17	Sharma et al. (24)	Increased	50	30	1.84 ± 0.81, *P* < 0.05	0.03 ± 0.02	33.84 ± 6.816	33.20 ± 7.014	India
IL-17	Hoşgören-Tekin et al. (31)	No association	31	56	45.92 (29.37–339.67)/NA	45.36 (16.69–291.21)	NA	NA	Turkey
IL-17A	Gora et al. (22)	Increased	32	40	41.4 (38.55–48.25)/<0.001	27.13 (20.37–36.45)	11.21 (8–14.42)	11 (7–14)	Poland
IL-17A	Zheng et al. (42)	No association	28	28	NA/>0.05	NA	35.6 ± 6.1	35.8 ± 7.9	China
IL-17F	Chen et al. (6)	No association	60	15	17.9 (0.0–55.1)/NA	106.8 (0.5–229.7)	NA	24.6 (22–29)	China
IL-21	Chen et al. (6)	Increased	60	15	1,063.5 (581.9–1,825.2)/0.012	562.8 (481.1–1,083.1)	NA	24.6 (22–29)	China
IL-22	Chen et al. (6)	No association	60	15	861.6 (503.0–2,085.5)/NA	589.8 (252.4–1,009.5)	NA	24.6 (22–29)	China
IL-23	Degirmenci et al. (39)	Decreased	40	20	NA/0.01	NA	38.2 ± 10.4	36.5 ± 5.45	Turkey
IL-23	Chen et al. (6)	Increased	60	15	306.3 (206.4–641.2)/0.038	212.1 (47.3–372.0)	NA	24.6 (22–29)	China
IL-23	Atwa et al. (33)	Increased	75	30	38.95 ± 27.82, *P* < 0.001	9.87 ± 4.62	31.8 ± 10.3	29.7 ± 9.1	Egypt
IL-23	Sharma et al. (24)	Increased	50	30	25.57 ± 10.79, *P* < 0.05	0.15 ± 0.14			
IL-23	Gora et al. (22)	Decreased	32	40	361.5 (289.0–364.9)/<0.001	603.0 (527.5–674.0)	11.21 (8–14.42)	11 (7–14)	Poland
IL-25	Băruta et al. (61)	Increased	50	33	140.27 ± 100.16, *P* = 0.0823	105.03 ± 89.21	50.14 ± 16.10	44.32 ± 9.23	Romania

Yr, year; Yrs, years; SD, standard deviation; CU, chronic urticaria.

### 3.4 Quality assessment

Assessment of study quality using the NHLBI Quality Assessment Tool for Case-Control Studies indicated that 44 of the 50 included studies (88%) were rated as high quality with a low risk of bias, while the remaining 6 studies (12%) were considered to have a moderate risk of bias (Table 4).

**Table 4:** Quality assessment.

Number	Quality Assessment: Case. Control studies (https://www.nhlbi.nih.gov/health-topics/study-quality-assessment-tools)													Abstract (fair)
	Author year	1	2	3	4	5	6	7	8	9	10	11	12	Overall quality rating - good, fair, and poor
1	Degirmenc (39)	y	y	NR	y	Y	y	y	NA	NA	y	y	NA	Good
2	Chen (6)	y	y	NR	y	y	y	y	NA	NA	y	y	NA	Good
3	Hoşgören-Tekin (31)	y	y	NR	y	y	y	y	NA	NA	y	y	NA	Good
4	Bae (54)	y	y	NR	y	y	y	y	NA	NA	y	y	NA	Good
5	Ferrer (40)	y	y	NR	y	y	y	y	NA	NA	y	y	NA	Good
6	Caproni (37)	y	y	NR	y	y	y	y	NA	NA	y	y	NA	Good
7	Trinh (32)	y	y	NR	y	y	y	y	NA	NA	y	y	NA	Good
8	Góra (22)	y	y	NR	y	y	y	y	NA	NA	y	y	NA	Good
9	Ucmak (43)	y	y	NR	y	y	y	y	NA	NA	y	y	NA	Good
10	Najjar (44)	y	y	NR	y	n	y	y	NA	NA	y	y	NA	Fair
11	Grieco (19)	y	y	NR	y	y	y	y	NA	NA	y	y	NA	Good
12	Kasperska-Zajac (45)	y	y	NR	y	y	y	y	NA	NA	y	y	NA	Good
13	Grzanka (46)	y	y	NR	y	y	y	y	NA	NA	y	y	NA	Good
14	Valerieva (47)	y	y	NR	y	n	y	y	NA	NA	y	y	NA	Fair
15	Kasperska-Zajac (48)	y	y	NR	y	y	y	y	NA	NA	y	y	NA	Good
16	Kasperska-Zajac (49)	y	y	NR	y	y	y	y	NA	NA	y	y	NA	Good
17	Kasperska-Zajac (50)	y	y	NR	y	y	y	y	NA	NA	y	y	NA	Good
18	Rajappa (51)	y	y	NR	y	y	y	y	NA	NA	y	y	NA	Good
19	Alasandagutti (38)	y	y	NR	y	y	y	y	NA	NA	y	y	NA	Good
20	Rasool (23)	y	y	NR	y	y	y	y	NA	NA	y	y	NA	Good
21	GRZANKA (52)	y	y	NR	y	y	y	y	NA	NA	y	y	NA	Good
22	Bhatia (53)	y	n	NR	y	y	n	y	NA	NA	y	y	NA	Good
23	ZHENG (42)	y	y	NR	y	y	y	y	NA	NA	y	y	NA	Good
24	Mohamed (41)	y	y	NR	y	n	y	y	NA	NA	y	y	NA	Fair
25	Piconi (18)	y	y	NR	y	y	y	y	NA	NA	y	y	NA	Good
26	Moos (25)	y	y	NR	y	y	y	y	NA	NA	y	y	NA	Good
27	Ene (21)	y	y	NR	y	y	y	y	NA	NA	y	y	NA	Good
28	Petrola (20)	y	y	NR	y	n	y	y	NA	NA	y	y	NA	Fair
29	Özçeker (63)	y	y	NR	y	y	y	y	NA	NA	y	y	NA	Good
30	Moghadam (64)	y	y	NR	y	y	y	y	NA	NA	y	y	NA	Good
31	Santos (17)	y	y	NR	y	y	y	y	NA	NA	y	y	NA	Good
32	Lin (56)	y	y	NR	y	y	y	y	NA	NA	y	y	NA	Good
33	Atwa (33)	y	y	NR	y	y	y	y	NA	NA	y	y	NA	Good
34	Grzanka (65)	y	y	NR	y	y	y	y	NA	NA	y	y	NA	Good
35	Sharma (24)	y	y	NR	y	y	y	y	NA	NA	y	y	NA	Good
36	Varghese (26)	y	y	NR	y	y	y	y	NA	NA	y	y	NA	Good
37	Kurt (27)	y	y	NR	y	y	y	y	NA	NA	y	y	NA	Good
38	Abdel-Bary (28)	y	y	NR	y	y	y	y	NA	NA	y	y	NA	Good
39	Tedeschi (29)	y	y	NR	y	y	y	y	NA	NA	y	y	NA	Good
40	Puxeddu (30)	y	y	NR	y	y	y	y	NA	NA	y	y	NA	Good
41	Montjoye (55)	y	y	NR	y	n	y	y	NA	NA	y	y	NA	Fair
42	Boyvadoglu (57)	y	y	NR	y	y	y	y	NA	NA	y	y	NA	Good
43	Băruta (58)	y	y	NR	y	y	y	y	NA	NA	y	y	NA	Good
44	Raap (59)	y	y	NR	y	y	y	y	NA	NA	y	y	NA	Good
45	Chaowattanapanit (60)	y	y	NR	y	y	y	y	NA	NA	y	y	NA	Good
46	Kulumbegov (62)	y	y	NR	y	y	y	y	NA	NA	y	y	NA	Good
47	Dobrican-Băruta (61)	y	y	NR	y	y	y	y	NA	NA	y	y	NA	Good
48	Bostan (34)	y	y	NR	y	y	y	y	NA	NA	y	y	NA	Good
49	Tedeschi (35)	y	y	NR	y	y	y	y	NA	NA	y	y	NA	Good
50	Grzanka (36)	y	y	NR	y	n	y	y	NA	NA	y	y	NA	Fair

## 4 Discussion

To our knowledge, this systematic review represents the first registered evaluation of serum Th1, Th2, and Th17 cytokines in patients with CU. Our meta-analysis revealed statistically significant pooled SMDs values for serum TNF-α and IL-17 levels. These findings may influence the clinical practice for CU patients, as measurements of serum TNF-α and IL-17 levels are accessible to some clinicians.

Tumor necrosis factor alpha (TNF-α) plays a critical role in inflammation, immune regulation, and apoptosis (36). It is released by human skin mast cells and other inflammatory cells present at urticarial lesion sites (66), making it a candidate mediator in urticaria pathogenesis (34). Our meta-analysis revealed a significant association between CU and elevated serum TNF-α levels, with a pooled SMD of 1.40 (95% CI of 0.59–2.21). This significant link confirms the autoimmune and inflammatory nature of CU, and supports the inclusion of TNF-α in the clinical assessment of CU.

IL-17, produced by T helper (Th) type 17 cells, binds to IL-17 receptors on epithelial, endothelial, and fibroblastic stromal cells (63). IL-17 is associated with many autoimmune disorders, such as psoriasis, multiple sclerosis, inflammatory bowel disease, rheumatoid arthritis, and asthma (67–69). Serum IL-17 levels were significantly elevated in CU patients compared to controls and correlated with urticaria severity (24, 33, 70) Our meta-analysis confirmed this, showing a significant pooled SMD of 1.43 (95% CI of 0.11–2.75). These findings support IL-17 as a valuable biomarker for CU clinical assessment.

A limitation of this systematic review and meta-analysis is the heterogeneity in assay methods. Variability in laboratory methods across studies (e.g., ELISA, multiplex assays) may lead to variations in cytokine measurements. Furthermore, data from some studies could not be pooled because of non-normal data distribution. In addition, owing to the limited number of related studies, we did not differentiate between chronic induced urticaria and chronic spontaneous urticaria in this review despite their distinct pathogenic mechanisms. The lack of subgroup analysis might restrict the clinical applicability of our findings.

Despite these limitations, the results of this review and meta-analysis provide high-quality evidence-based insights into the associations between serum Th1, Th2, and Th17 cytokines and CU. In conclusion, Th1 and Th17-derivated cytokines play crucial roles in the pathogenesis of CU. CU is thus characterized as a chronic inflammatory state mediated by diverse cytokines and autoimmune mechanisms. These findings may help inform clinical guidelines for CU diagnosis by incorporating serum TNF-α and IL-17 levels. However, the clinical cut-off values, sensitivity, specificity, and practical implementation of these biomarkers have not yet been explored. Therefore, well designed and large-scale studies are needed to determine the optimal cutoff levels of TNF-α and IL-17.
