# Supplementary material for: The roles of serum Th1, Th2, and Th17 cytokines in patients with chronic urticaria: a systematic review and meta-analysis
Source: Front Allergy. 2025 Oct 3;6:1673041. doi: 10.3389/falgy.2025.1673041 (PMC12531172; doi:10.3389/falgy.2025.1673041)
Supplement: Supplementary file 1 [file Table1.docx]

**Supplementary Table 1- Summary of studies included in the review**

| Number | Study (author-yr) | Title | country |
| --- | --- | --- | --- |
| 1 | Degirmenc  2016[46] | Analysis of the association of chronic spontaneous urticaria with interlekin-4, -10, transforming growth factor-b1, interferon-γ, interleukin-17A and -23 by autologous serum skin test | Turkey |
| 2 | Chen2017[29] | IL4/13-Different expression patterns of plasma Th1‐, Th2‐, Th17‐ and Th22‐related cytokines correlate with serum autoreactivity and allergen sensitivity in chronic spontaneous urticaria | China |
| 3 | Hoşgören-Tekin2024[41] | Infammatory cytokine levels and changes during omalizumab  treatment in chronic spontaneous urticaria | Turkey |
| 4 | Bae2016[61] | Periostin and Interleukin-13 Are Independently Related to Chronic Spontaneous Urticaria | Korea |
| 5 | Ferrer2002[47] | Secretion of Cytokines, Histamineand Leukotrienes in Chronic Urticaria | USA |
| 6 | Caproni2004[44] | Serological detection of eotaxin, IL-4, IL-13, IFN-g, MIP-1a, TARC and IP-10 in chronic autoimmune urticaria and chronic idiopathic urticaria | Italy |
| 7 | Trinh2016[42] | Altered Systemic Adipokines in Patients with Chronic Urticaria | South Korea |
| 8 | Góra2022[33] | Assessment of selected interleukins (IL-6, IL-17A, IL-18, IL-23) and chemokines (RANTES, IP-10) in children with acute and chronic urticaria | Poland |
| 9 | Ucmak2013[50] | Determination of dermatology life quality index, and serum C-reactive protein and plasma interleukin-6 levels in patients with chronic urticaria | Turkey |
| 10 | Najjar2018[51] | Hyperlipidemia in association with pro-inflammatory cytokines among chronic spontaneous urticaria Case-control study | Egypt |
| 11 | Grieco2020[30] | IFN-γ/IL-6 and related cytokines in chronic spontaneous urticaria evaluation of their pathogenetic role and changes during omalizumab therapy | Italy |
| 12 | Kasperska-Zajac2015[52] | IL-6 Transsignaling in Patients with Chronic Spontaneous Urticaria | Poland |
| 13 | Grzanka2018[53] | Interplay between acute phase response and coagulation/fibrinolysis in chronic spontaneous urticaria | Poland |
| 14 | Valerieva2017[54] | IL6- Markers for systemic inflammation (CRP, ESR, IL-6, IL-10, IL-33, CCL2/MCP-1), and the disease severity in patients with chronic spontaneous urticaria | Bulgaria |
| 15 | Kasperska-Zajac2013[55] | Markers of systemic inflammation in delayed pressure urticaria | Poland |
| 16 | Kasperska-Zajac2007[56] | Plasma concentration of interleukin 6 (IL-6), and its relationship with circulating concentration of dehydroepiandrosterone sulfate (DHEA-S) in patients with chronic idiopathic urticaria | Poland |
| 17 | Kasperska-Zajac2011[57] | Plasma IL-6 concentration correlates with clinical disease activity and serum C-reactive protein concentration in chronic urticaria patients | Poland |
| 18 | Rajappa2013[58] | Platelet oxidative stress and systemic inflammation in chronic spontaneous urticaria | India |
| 19 | Alasandagutti2014[45] | Role of IFN-γ and IL-6 Cytokines and Their Association in Determining Susceptibility to Chronic Idiopathic Urticaria | India |
| 20 | Rasool2014[34] | Study of serum interleukin (IL) 18 and IL-6 levels in relation with the clinical disease severity in chronic idiopathic urticaria patients of Kashmir (North India) | India |
| 21 | GRZANKA2015[59] | Systemic inflammatory response and calcification markers in patients with long lasting moderate-severe chronic spontaneous urticaria | Poland |
| 22 | Bhatia2024[60] | A prospective observational study correlating possible novel biomarkers with disease severity and antihistamine response in chronic spontaneous urticaria | India |
| 23 | ZHENG2017[49] | Analysis of the changes in Th9 cells and related cytokines in the peripheral blood of spontaneous urticaria patients | China |
| 24 | Mohamed2003[48] | Increased circulating FcepsilonRIl-bearing B- lymphocytes and serum levels of IL-4 in non- autoreactive chronic idiopathic urticaria | Egypt |
| 25 | Piconi2001[28] | Immune Profiles of Patients with Chronic Idiopathic Urticaria | Italy |
| 26 | Moos2021[35] | The Role of Interleukin 10 and 18 in Chronic Spontaneous Urticaria Pathogenesis in the Context of Angioedema Coexistence | Poland |
| 27 | Ene2024[32] | Changes in Serum IL-12 Levels Following the Administration of H1-Antihistamines in Patients with Chronic Spontaneous Urticaria | Romania |
| 28 | Petrola2009[31] | Serum Th1 and Th2 Cytokines in Chronic Urticaria Patients | Venezuela |
| 29 | Özçeker2024[70] | Do Cytokines Play a Role in Chronic Spontaneous Urticaria in Childhood? IL-17 One of Them? | Turkey |
| 30 | Moghadam2017[71] | Evaluation of IL-17 serum level in patients with chronic idiopathic urticaria based on the autologous skin test compared to the control group | Iran |
| 31 | Santos2008[27] | Increased circulating pro-inflammatory cytokines and imbalanced regulatory T-cell cytokines production in chronic idiopathic urticaria | Brazil |
| 32 | Lin2017[63] | Increased plasma IL-17, IL-31, and IL-33 levels in chronic spontaneous urticaria | China |
| 33 | Atwa2014[25] | Serum concentration of IL-17, IL-23 and TNF-α among patients with chronic spontaneous urticaria association with disease activity and autologous serum skin test | Egypt |
| 34 | Grzanka2017[72] | The relationship between circulating concentrations of interleukin 17 and C reactive protein in chronic spontaneous urticaria | Poland |
| 35 | Sharma2020[26] | To evaluate the role and relevance of cytokines IL-17, IL-18, IL-23 and TNF-α and their correlation with disease severity in chronic urticaria | India |
| 36 | Varghese2016[36] | Association among stress, hypocortisolism, systemic inflammation, and disease severity in chronic urticaria | India |
| 37 | Kurt2011[37] | Autologous serum skin test response in chronic spontaneous urticaria and respiratory diseases and its relationship with serum interleukin-18 level | Turkey |
| 38 | Abdel-Bary2021[38] | Serum interleukin-18 and immunoglobulin E in chronic spontaneous urticaria and their relation to severity of the disease | Egypt |
| 39 | Tedeschi2007[39] | Serum interleukin-18 in patients with chronic ordinary urticaria association with disease activity | Italy |
| 40 | Puxeddu2013[40] | Study on the levels of IL-18, IL-33 and IgE in patients with chronic urticaria | Italy |
| 41 | Montjoye2019[62] | Increased expression of IL-24 in chronic spontaneous urticaria | Belgium |
| 42 | Boyvadoglu2023[64] | Effects of Omalizumab on Serum Levels of Substance P, Calcitonin Gene-Related Peptide, Neuropeptide Y, and Interleukin-31 in Patients with Chronic Spontaneous Urticaria | Turkey |
| 43 | Dobrican-Băruta  2023[65] | IL-31-Pruritus Interleukin Serum Values and Clinical Impact in Chronic Spontaneous Urticaria-A Romanian Retrospective Study | Romania |
| 44 | Raap2010[66] | Increased levels of serum IL-31 in chronic spontaneous urticaria* | Germany |
| 45 | Chaowattanapanit2020[67] | Increased serum IL-31 levels in chronic spontaneous urticaria and psoriasis with pruritic symptoms | Thailand |
| 46 | Kulumbegov2023[69] | Interleukin-33, endothelin-1, and inflammatory parameters in chronic spontaneous urticaria | Georgia |
| 47 | Dobrican-Băruta2024[68] | The Alarmin Triad-IL-25, IL-33, and TSLP-Serum Levels and Their Clinical Implications in Chronic Spontaneous Urticaria | Romania |
| 48 | Bostan2023[19] | Cytokine Profiles of Chronic Urticaria Patients and The Effect of Omalizumab Treatment | Turkey |
| 49 | Tedeschi2006[43] | No evidence of tumor necrosis factor-alpha release in blood of patients with chronic urticaria | Italy |
| 50 | Grzanka2019[73] | Tumor necrosis factor-alpha and Fas/Fas ligand signaling pathways in chronic spontaneous urticaria | Poland |

Yr- year, Yrs-years, SD- standard deviation, C**U- chronic urticaria**
